# A computer-designed scaffold for bone regeneration within cranial defect using human dental pulp stem cells

**DOI:** 10.1038/srep12721

**Published:** 2015-08-03

**Authors:** Doo Yeon Kwon, Jin Seon Kwon, Seung Hun Park, Ji Hun Park, So Hee Jang, Xiang Yun Yin, Jeong-Ho Yun, Jae Ho Kim, Byoung Hyun Min, Jun Hee Lee, Wan-Doo Kim, Moon Suk Kim

**Affiliations:** 1 Department of Molecular Science and Technology, Ajou University, Suwon 443-759, Korea; 2 Nature-Inspired Mechanical System Team, Korea Institute of Machinery and Materials, Daejeon 305-343, Korea; 3 Department of Dentistry, School of Medicine, Inha University, Incheon 440-711, Korea

## Abstract

A computer-designed, solvent-free scaffold offer several potential advantages such as ease of customized manufacture and *in vivo* safety. In this work, we firstly used a computer-designed, solvent-free scaffold and human dental pulp stem cells (hDPSCs) to regenerate neo-bone within cranial bone defects. The hDPSCs expressed mesenchymal stem cell markers and served as an abundant source of stem cells with a high proliferation rate. In addition, hDPSCs showed a phenotype of differentiated osteoblasts in the presence of osteogenic factors (OF). We used solid freeform fabrication (SFF) with biodegradable polyesters (MPEG-(PLLA-*co*-PGA-*co*-PCL) (PLGC)) to fabricate a computer-designed scaffold. The SFF technology gave quick and reproducible results. To assess bone tissue engineering *in vivo*, the computer-designed, circular PLGC scaffold was implanted into a full-thickness cranial bone defect and monitored by micro-computed tomography (CT) and histology of the *in vivo* tissue-engineered bone. Neo-bone formation of more than 50% in both micro-CT and histology tests was observed at only PLGC scaffold with hDPSCs/OF. Furthermore, the PLGC scaffold gradually degraded, as evidenced by the fluorescent-labeled PLGC scaffold, which provides information to tract biodegradation of implanted PLGC scaffold. In conclusion, we confirmed neo-bone formation within a cranial bone defect using hDPSCs and a computer-designed PLGC scaffold.

Engineering new bone tissue to repair and regenerate bone at bone defect sites represents one of the most challenging emergent fields^1^. Success of the procedure is determined by use of a suitable scaffold, a structural support for osteoprogenitor cells, and osteoinductive factors necessary to regenerate neo-bone at the site of bone defect^2^.

In general, bone defects are irregularly shaped. Therefore, the scaffold should allow for complicated and irregular bone geometry^3^. The scaffold consists of three-dimensional (3D) interconnected pores that allow for uniform penetration of nutrients and removal of metabolic waste *in vivo* to assist the development of new bone tissue^4^. In addition, the scaffold must also be biodegradable to keep pace with the formation of the new bone tissue^5^. Therefore, an ideal scaffold for bone tissue engineering should possess bone-like geometry and a 3D porous structure for bone defects, as well as be biodegradable to match the formation of a neo-bone tissue^6^,^7^,^8^,^9^.

Several scaffold fabrication techniques can be utilized to meet the requirements of the target bone defect. However, the production of a scaffold with complicated and irregular geometry can be limited by conventional scaffold fabrication techniques^10^. Therefore, recent research efforts have focused on the fabrication of a scaffold with complicated and interconnected pore structure, including several computer-designed scaffold fabrication techniques; for example, solid freeform fabrication (SFF)^11^,^12^,^13^. These pioneering techniques can be used to fabricate a customized and solvent-free scaffold with tailed bone geometry and interconnected pore structures for repair of bone defects.

SFF techniques require selection of an appropriate biomaterial for the scaffold, with biocompatible, safe, and non-immunogenic properties. Several biomaterials are available, including not only biodegradable polyesters such as poly-L-lactide (PLLA), polyglycolide (PGA) (or their PLGA co-polyesters), and polycaprolactone (PCL) but also natural polymers such as collagen, gelatin, alginate^14^,^15^,^16^,^17^,^18^. Among several polyester biomaterials, PCL are one of the most widely used polyesters as a principal biomaterial in SFF, however PCL alone is limited by slow degradation kinetics (2–4 years).

In general, the degradation of scaffolds fabricated from PLLA, PGA, and PCL or their co-polyesters can be controlled by attributes such as the molecular weight and composition of polyesters^19^. The degradation of the fabricated scaffold occurs via scission of ester bond linkages in the polymer backbone via hydrolytic attack by water molecules over a period ranging from days to a few weeks. Our previous work demonstrated the feasibility of PLGC block copolymers with various proportions of PLLA, PGA, and PCL as printing materials for SFF^20^,^21^. Thus we thought that MPEG-(PLLA-*co*-PGA-*co*-PCL) (PLGC) copolymer is a suitable biomaterial for SFF, ideally tailored to match the bone regeneration rate of up to 12 weeks, as PLGC showed half-life of molecular weight of 8 weeks.

During the SFF method of fabrication, the scaffold must be prepared by changing from a flowable liquid to a solid form, without the use of organic solvents, at ambient temperature after continuous deposition by SFF^14^,^22^. Thus the *first aim of this work* was the fabrication of a computer-designed and solvent-free PLGC scaffold using SFF to provide several potential advantages such as ease of customized manufacture and *in vivo* safety because there was no study regarding to utilization of PLGC scaffold for cranial bone defect using SFF.

Recent progress in studies of stem cells serves as potential viable therapeutic options for patients. Among several stem cells, the human bone marrow stem cells (hBMSCs) have provided valuable leading clinical data^23^. However, the major issue of hBMSCs is that it requires a painful surgical procedure to harvest them. Furthermore, the yields of harvested hBMSCs are usually low.

Thus, it requires *ex vivo* culturing and expanding process to supply a higher number of hBMSCs for the clinical application. This process requires strict compliance with good medical practice to completely eliminate the risk of contamination during expansion^23^,^24^.

Human dental pulp stem cells (hDPSCs) can be easily harvested in large quantities from discarded extract teeth during dental surgery. This can cause patients to experience little pain and thus is a very common, safe, and effective procedure. In addition, hDPSCs have clonogenic ability with a rapid proliferation rate, and can be cryopreserved for long periods^25^,^26^. These considerations make hDPSCs more attractive for clinical studies.

More importantly, hDPSCs are also stem cells, capable of multipotent or multilineage differentiations, showing the potential to differentiate into other cell lineages such as odontoblastic, neurogenic, and adipocytic cell types. In addition, hDPSCs could be a desirable option as a source of cells for bone tissue engineering due to their distinct ability to differentiate into the osteogenic lineage. Although hDPSCs could be a promising source of stem cells with characteristics such as accessibility, rapid proliferation rate, and the ability to differentiate into osteogenic cells in bone tissue engineering^27^,^28^,^29^,^30^,^31^,^32^,^33^, to the best of our knowledge, there was no or little study regarding to utilization of hDPSCs in bone tissue engineering using SFF fabricated PLGC scaffold. Thus, the *second aim of this work* was to show that hDPSCs can act as a cell source of SFF fabricated PLGC scaffold for cranial bone defect.

The basic approach of bone tissue engineering includes creating neo-bone tissue within implanted biodegradable polymeric scaffolds with osteoprogenitor cells *in vitro* and/or *in vivo*. To date, extensive research has been performed to monitor scaffold degradation and bone regeneration by sacrifice of test animals at various time points^34^,^35^. This method often leads to inaccurate conclusions owing to variations between animals and because the rate of scaffold degradation needs to be correlated with that of neo-bone tissue formation over time. Few studies have examined the *in vivo* correlation between scaffold degradation and formation of neo-bone tissue^36^. Based on this concept, the *third aim of this work* was to understand whether SFF fabricated PLGC scaffold degraded over the appropriate experimental time frame to guide the formation of neo-bone tissue into the cranial bone defects.

To the best of our knowledge, the overall current study was first study regarding to utilization of a computer-designed, solvent-free PLGC scaffold and hDPSCs for neo-bone regeneration within cranial bone defect (Fig. 1). We sought answers to the following specific questions: 1) Can hDPSCs serve as a new source of cells for computer-designed and solvent-free PLGC scaffold in bone tissue engineering? 2) Does *in vivo* neo-bone formation occur in response to a PLGC scaffold seeded with hDPSCs and osteogenic factors (OF)? 3) Can the correlation of scaffold degradation and neo-bone ingrowth be monitored by using a fluorescent-labeled PLGC scaffold? Answers to these questions will have implications for the application of hDPSCs and computer-designed PLGC scaffolds in bone tissue engineering.

## Results

### Characterization of hDPSCs

The hDPSCs, isolated from a young woman, proliferated rapidly in culture medium and were cultured up to 5 passages. The expression of surface antigens by hDPSCs was evaluated using flow cytometry. hDPSCs at passage 5 were positive for the mesenchymal stem cell markers CD90 (>99.3%), CD105 (>95.6%), and CD166 (99.4%), and negative for the hematopoietic stem cell markers CD34 (<0.01%) and CD45 (<0.01%). hDPSCs maintained stem cell characteristics through passages 2 to 5.

To examine the proliferative potential of hDPSCs as a source of cells, cell counts were performed at passages 2, 3, 4, and 5. hDPSCs quickly expanded with subsequent passages. The hDPSCs proliferated from [1 × 10^6^] to [3.68 (±0.96) × 10^6^]*, [5.67 (±0.95) × 10^6^]*, [6.73 (±2.16) × 10^6^]*, and [4.55 (±0.35) × 10^6^]* cells at passage 2, 3, 4, and 5, respectively (*p > 0.5). Proliferation of hDPSCs was approximately 3 times greater than that of hBMSCs at the same passage (Fig. S1). hDPSCs are thus not only a more abundant source of cells than hBMSCs, but also have a higher proliferation rate. These results indicate that hDPSCs could be used as a new source of multipotent stem cells in the experiments to follow.

### *In vitro* osteogenic differentiation of hDPSCs

Osteogenic differentiation of hDPSCs was investigated in culture plates with osteogenic factors (OF) (a mixture of β-glyceraldehyde-3-phosphate, l-ascorbic acid, dexamethasone, and rhBMP-2) (Fig. S2). Osteogenic characteristics were identified by ALP, ARS, and VK staining. ALP staining produced a faint black color at 2 weeks, which became black at 3 and 4 weeks. hDPSCs exhibited faint red ARS staining at 2 weeks, which became more widely distributed and deep after 3 and 4 weeks. VK staining gave a brown color at 2 weeks, indicating calcium deposition. The staining darkened and became more widely distributed after 3 and 4 weeks. These data provide evidence of osteogenic differentiation by hDPSCs in the presence of OF.

ALP contents were determined to compare the osteogenic differentiation of hDPSCs with hBMSCs at 1 and 7 days (Fig. S3). ALP contents of hDPSCs were significantly higher than those of hBMSCs at 1 and 7 days, respectively (*p *< 0.001). These results demonstrated that the hDPSCs are capable of differentiation into the osteogenic lineage, properties that effectively define as a promising source of stem cells in bone tissue engineering.

### Fabrication of circular PLGC scaffold

**Figure 2:**
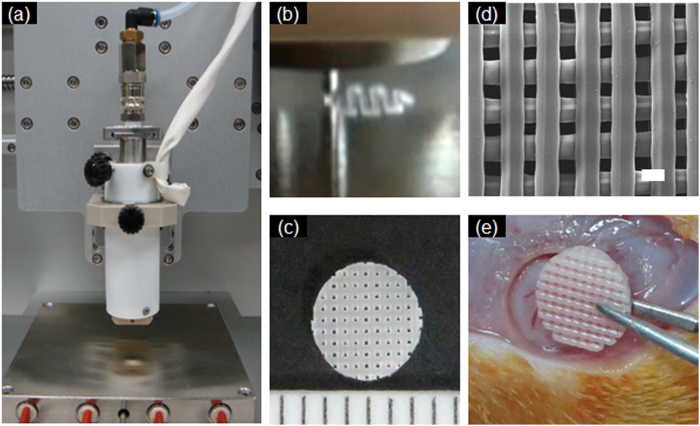
(**a,b**) Fabrication process of the PLGC scaffold using a solid free-form fabrication method, (**c**) optical microscope images, (**d**) SEM image (scale bar: 300 μm) and (**e**) implantation of the fabricated PLGC scaffold.

Figure 2 panels ***a*** through ***b*** show the fabrication process for the circular PLGC scaffold. PLGC exhibited good mobility from the micronozzle for 3D plotting and then solidified within an appropriate time period at ambient temperature, indicating that PLGC is a printable biomaterial for SFF.

The circular PLGC scaffold was plotted layer by layer with orthogonal orientation on stainless plates in a computer-designed circular form, by SFF. The fabricated circular PLGC scaffold was 5 mm in diameter, 1 mm in length, with a 300-μm strand size and a 200-μm pore size in the vertical cross-section (Fig. 2c,d) (Fig. S4).

The compressive modulus of the PLGC scaffold was measured to compare maintenance of the structure for 3 specimens. At a 50% compressive ratio, the compressive modulus of the PLGC scaffold was measured to be 49.3 ± 9.1 MPa.

The fabrication time of a single circular PLGC scaffold was around 3 min. Considering the fabrication of only circular PLGC scaffolds, SFF productivity is expected to be greater than that obtained by conventional, manual-based fabrication techniques.

### *In vitro* cell attachment and proliferation on fabricated PLGC scaffold

**Figure 3:**
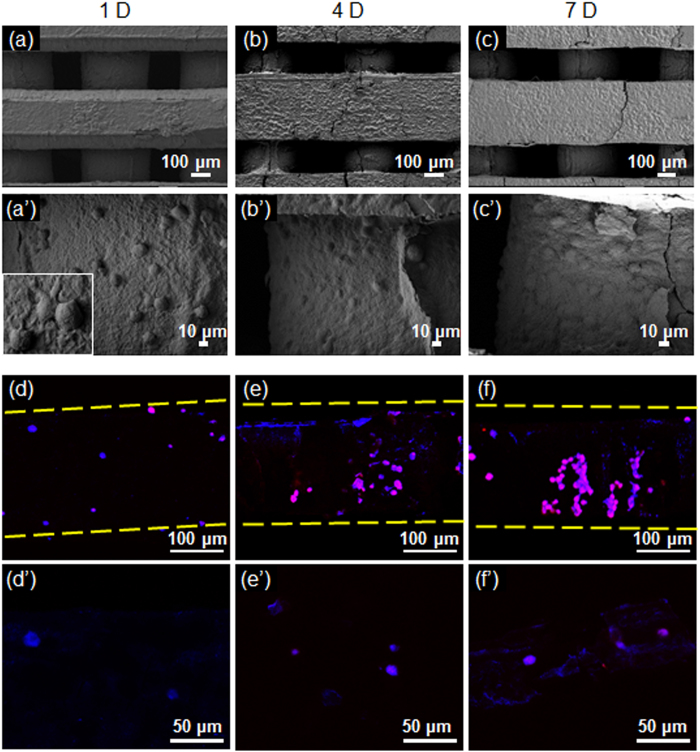
SEM (**a–c** and **a’–c’**) and fluorescent (**d–f** and **d’–f’**) images showing the hDPSCs on the PLGC scaffold at 1, 4 and 7 days. (**a–c**) 100 μm (100 × magnification) and (**a’–c’**) 10 μm (500 × magnification). The scale bars of fluorescent images represent (**d–f**) 100 μm (200 × magnification) and (**d’–f’**) 50 μm (400 × magnification).

The attachment and proliferation of hDPSCs on PLGC scaffold were monitored over a 7-day incubation period (Fig. 3). The hDPSCs appear on PLGC scaffold at 1 day. The majority of the attached hDPSCs were round in shape. High-magnification SEM images showed that by this time formation of cytoplasmic extensions was observed on PLGC scaffold. Their filopodia was anchored to the surface of PLGC scaffold (box image in Fig. 3a’). The hDPSCs spread out on PLGC scaffold at 5 and 7 days. The hDPSCs are uniformly and abundantly distributed in the whole area of PLGC scaffold surface.

Fluorescent images of hDPSCs on PLGC scaffold were obtained after culture over a 7-day period (Fig. 3). Blue fluorescence was indicative of Hoechst-stained nuclei. Actin showed as red fluorescence, while pink fluorescence was due to the overlay of the blue and red fluorescent images. The PLGC scaffold alone showed no fluorescent images. Meanwhile, the hDPSCs on PLGC scaffold showed increased blue fluorescence as a function of culture time. Pink fluorescence observed in the hDPSCs on PLGC scaffold in the merged images was increased with incubation time. These results imply that the hDPSCs were able to grow well on the PLGC scaffold surface. This indicates that the PLGC scaffold was suitably fabricated for hDPSCs, and used in the following *in vivo* experiment.

### *In vivo* implantation

To assess the *in vivo* tissue-engineered bone formation of the circular PLGC scaffold, surgeries were performed as shown in Fig. 2e. The PLGC scaffold alone or the PLGC scaffold/hDPSCs/OF was implanted into cranial bone to assess the extent of neo-bone formation. The implantation procedure as well as the implanted scaffold was well-tolerated in all models.

### *In vivo* degradation of PLGC scaffolds

To assess *in vivo* degradation, the PLGC scaffold alone or the PLGC scaffold/hDPSCs/OF was implanted into the cranial bone defects and then biopsied at different time points over 4, 8, and 12 weeks. The extent of degradation was monitored by GPC, fluorescence, and NMR.

**Figure 4:**
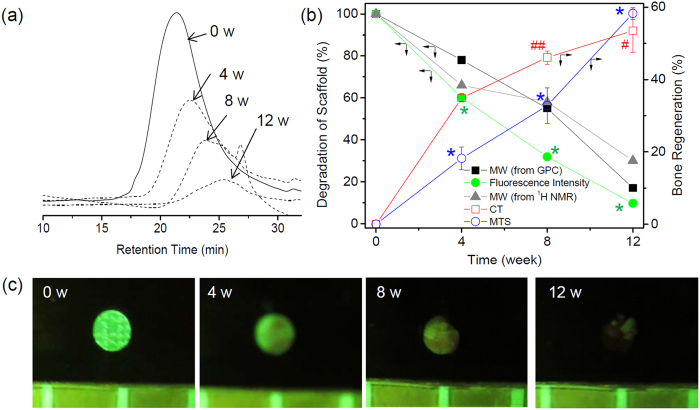
(**a**) GPC change graphs of the *in vivo*-degraded PLGC scaffold with hDPSCs/OF 0–12 weeks after implantation. (**b**) Implantation time versus the degraded molecular weight (measured from the maximum GPC peak (-◼-) and NMR (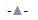) of *in vivo* degraded PLGC scaffold at 0–12 weeks after implantation, *p < 0.001, ^#^*p* < 0.005, ^##^*p* < 0.05) versus fluorescence intensity (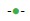) [calculated from (**c**)] and versus the regenerated bone volume (calculated from the micro-CT (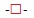) at Fig. 6 and Masson’s trichrome staining (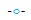) at Fig. 9). (c) Fluorescence images of the PLGC-FITC scaffold removed 0–12 weeks after implantation.

GPC traces of the PLGC scaffold alone and the PLGC scaffold/hDPSCs/OF showed a low-molecular-weight peak corresponding to a degraded species from 4 weeks (Fig. 4a). The PLGC scaffold alone and the PLGC scaffold/hDPSCs/OF showed a similar degraded GPC peaks (Fig. S5). After measuring the molecular weight of the *in vivo*-degraded PLGC scaffold alone and PLGC scaffold/hDPSCs/OF at the maximum GPC peak, the extent of degradation was calculated from the relative ratio of molecular weights determined during the experimental period and on the initial day. The intensity of the peaks decreased gradually as implantation time increased, indicating gradual degradation over time. The PLGC scaffold reached the degraded molecular weights of calc. 78%, 55%, and 17% of the original molecular weight at 4, 8, and 12 weeks, respectively (shown as a black line in Fig. 4b).

**Figure 5:**
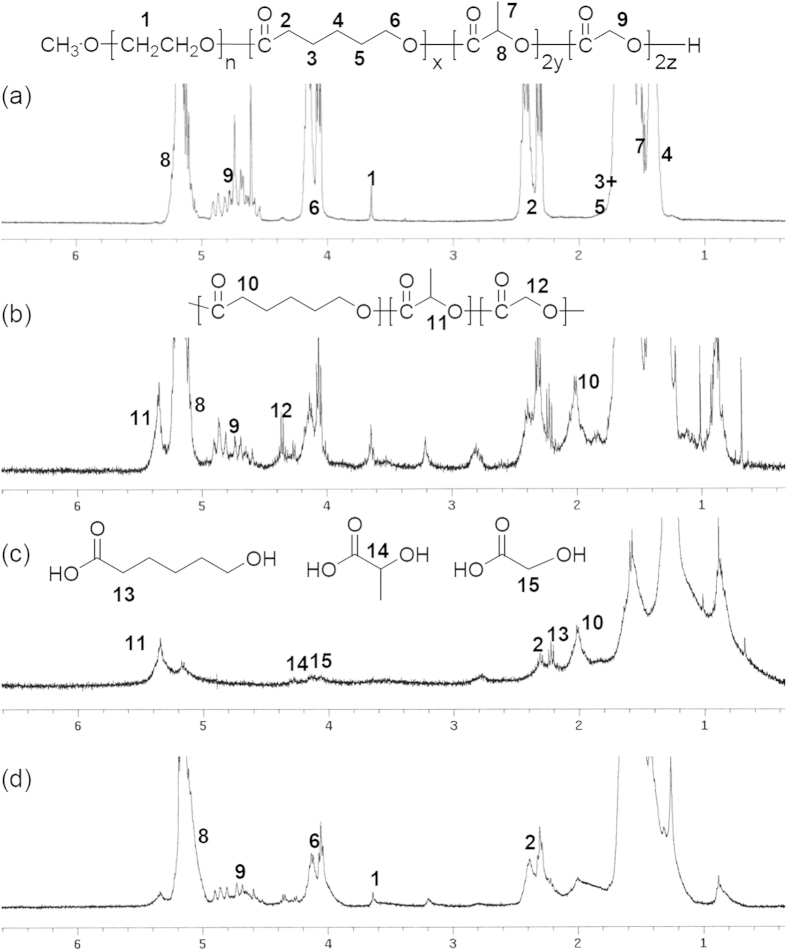
^1^H NMR spectra of the PLGC scaffold (**a**) before degradation and (**b**–**d**) after 8 weeks *in vivo*. (**b**) Crude mixture, (**c**) *n*-hexane and ethyl ether soluble portions, and (**d**) insoluble portions.

Figure 5 shows ^1^H-NMR spectral changes in the degraded PLGC scaffold before and after 8 weeks. The spectrum also shows that the characteristic peaks of degraded polymer and parent MPEG were obtained after separation in *n*-hexane and ether. Signals *14* and *11,* attributable to lactic acid, and signals *13* and *1*, attributable to 6-hydroxyl hexanoic acid and MPEG, respectively (Fig. 5b,c,d), appeared in the spectrum at 4.18 ppm, 1.14 ppm, 2.2 ppm, and 3.62 ppm, respectively.

The extent of degradation were determined by ^1^H NMR spectrometry though comparing the intensity of the methylene proton signals of MPEG, PCL, and PLLA at δ of 3.62, 4.0–4.2, and 5.0–5.25, respectively. The degraded molecular weights of PLGC scaffold were determined by ^1^H-NMR as calc. 66%, 58%, and 30% at 4, 8, and 12 weeks, respectively (shown as a gray line in Fig. 4b) (Fig. S6). The extent of *in vivo* degradation of PLGC scaffold well matches in GPC and NMR. Additionally, it was observed that the PLGC scaffolds degraded without significant difference in the absence and presence cells (Fig. S5).

### Confirmation of bone formation via micro-CT

**Figure 6:**
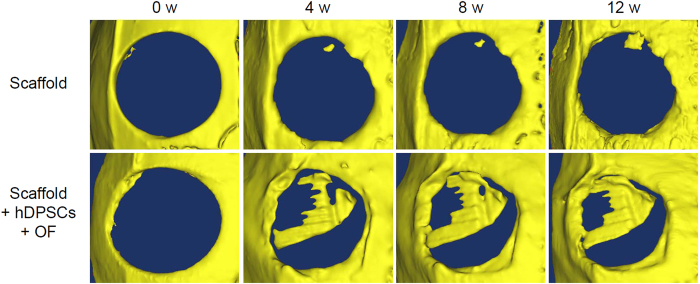
Virtual micro-CT images from rats receiving the PLGC scaffold without or with hDPSCs/OF at 0–12 weeks after implantation.

Generally, concerning bone scaffold, micro-CT has been widely employed to quantify bone mineral densities of newly formed bones^37^. Thus, in this work the ability of the PLGC scaffold to facilitate bone growth within a full-thickness bone defect was examined in rat cranial bone defects using the micro-CT. Each experimental model was analyzed at four time points: 0, 4, 8, and 12 weeks after PLGC scaffold implantation. Figure 6 shows the 3D micro-CT analysis, which revealed the changes in bone volume for the PLGC scaffold alone and the PLGC scaffold/hDPSCs/OF groups. The PLGC scaffold alone showed almost no view of bone-like ingrowths in the defective area except at the near original bone. Meanwhile, the view of the cranium of the PLGC scaffold/hDPSCs/OF at 4, 8, and 12 weeks post-implantation showed extensive bone-like ingrowths.

The extent of the neo-bone formation can be determined from the corresponding CT images (Fig. S7). There were no significant increases in the bone regeneration volumes of the PLGC scaffold alone. The bone regeneration of CT images demonstrated 1.5%, 2.4%, and 6% at 4, 8, and 12 weeks, respectively, for PLGC scaffold alone. Meanwhile in the case of the PLGC scaffold/hDPSCs/OF, bone regeneration increased from 0 to 35%, 46%, and 53% at 4, 8, and 12 weeks, respectively. This indicated that the defect area in the PLGC scaffold/hDPSCs/OF group was replaced by neo-bone tissues. The changes in the bone regeneration volume were determined after 0, 4, 8, and 12 weeks and plotted (shown as a red line in Fig. 4b).

### *In vivo* fluorescence imaging

The PLGC-FITC scaffold was fabricated by using fluorescent-labeled PLGC. The PLGC-FITC scaffold was removed after 0, 4, 8, and 12 weeks (Fig. 4c). Fluorescent images were obtained to monitor biodegradation of the PLGC-FITC scaffold. High levels of green fluorescence were observed at 0 weeks. The intensity of fluorescence gradually decreased. Negligible fluorescence was observed after 12 weeks, reaching a final intensity of 9% of the initial (shown as a green line in Fig. 4b). The change in the fluorescence intensity of PLGC-FITC scaffold images showed profiles similar to that of PLGC obtained by GPC and NMR, as shown in Fig. 4b. This result indicates that the degradation of PLGC gradually occurred *in vivo* and results from fluorescence imaging, GPC and NMR were consistent.

### Confirmation of bone formation via histology of *in vivo* tissue-engineered bone

**Figure 7:**
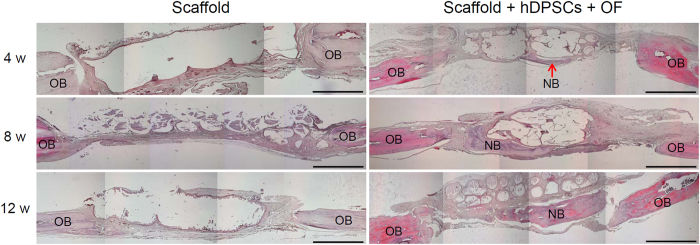
H&E staining of cranial bone for the PLGC scaffold without or with hDPSCs/OF at 0–12 weeks after implantation. Scale bar is 1 mm. (OB and NB indicate original bone and neo-bone, respectively).

After 4, 8, and 12 weeks, cranial tissue-engineered bone was removed, sectioned, and stained to compare the degree of neo-bone formation between the transverse sections (enlarged images in Figs S8,9,10). H&E staining indicated an empty central space with a clear border between the defective area and the original bone in the case of PLGC scaffold alone at 4, 8, and 12 weeks (Fig. 7). However, the stained images of the PLGC scaffold/hDPSCs/OF show an organized tissue structure between the defective area and the original bone at 4 weeks post implantation, which became more evident at later time points.

**Figure 8:**
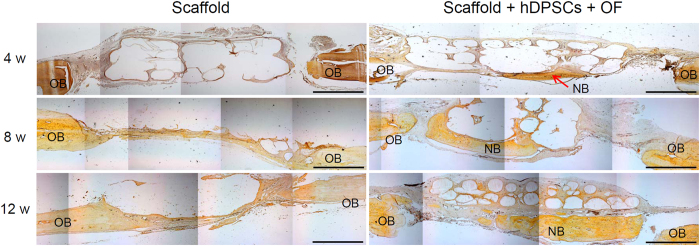
Von-Kossa staining of cranial bone for the PLGC scaffold, without or with hDPSCs/OF at 0–12 weeks after implantation. Scale bar is 1 mm. (OB and NB indicate original bone and neo-bone, respectively).

VK staining can help detect osteogenesis. PLGC scaffold alone showed little brown nodules between the defective area and the original bone at 4, 8, and 12 weeks (Fig. 8). Meanwhile, numerous brown nodules were evident in the PLGC scaffold/hDPSCs/OF and these increased with time.

**Figure 9:**
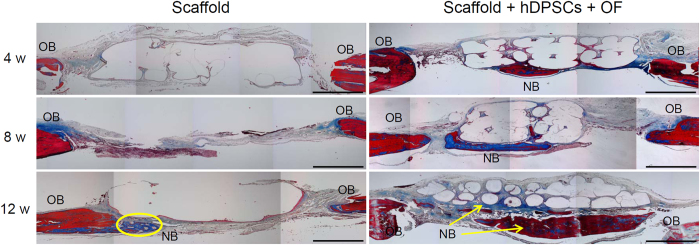
Masson’s trichrome staining of cranial bone for the PLGC scaffold, without or with hDPSCs/OF at 0–12 weeks after implantation. Scale bar is 1 mm. (OB and NB indicate original bone and neo-bone, respectively).

MTS staining showed evidence of collagen formation (blue) in part of the repaired area in PLGC scaffold/hDPSCs/OF (Fig. 9). Collagen matrix staining of neo-bone tissue increased markedly in the 8 and 12 weeks PLGC scaffold/hDPSCs/OF specimens. In contrast, MTS could not detect new collagen matrix deposition in any of the sites implanted with PLGC scaffold alone except at the border between the defective area and the original bone.

The semi-quantitative measurements of the traverse sections of MTS staining demonstrated 2%, 4%, and 8% blue stained area at 4, 8, and 12 weeks, respectively, for PLGC scaffold alone (Fig. S11). The MTS positive image was probably due to little tissue structure between the defective area and the original bone.

However, the sections of PLGC scaffold/hDPSCs/OF exhibited 18%, 33%, and 58% bone healing at 4, 8, and 12 weeks, respectively (shown as a blue line in Fig. 4b). This result clearly demonstrates the presence of neo-bone tissue with a typical mature bone structure *in vivo*, within the defect in the PLGC scaffold/hDPSCs/OF group. In addition, results from histological imaging and micro-CT were consistent.

The extent of remained scaffold areas was determined in histology images of H&E, VK and MTS staining and calculated as the relative ratio of the non-degraded PLGC scaffold during the experimental period and on the initial day (Fig. S12). The extent of the areas decreased gradually as implantation time increased. The decreasing profiles well matched with the degraded molecular weights of PLGC scaffold in GPC and NMR. This indicated that the volume initially occupied by the SFF fabricated PLGC scaffold degraded over the appropriate experimental time frame.

## Discussion

In recent years, bone tissue engineering has emerged as a promising approach to restore bone defects with osteoprogenitor cells and scaffolds that can be implanted *in vivo* at the site of bone defect^1^.

hDPSCs are easily harvested by comparatively non-invasive surgery without any side effects or inconvenience to patients, making it easy to obtain a large quantity of hDPSC^25^,^26^. Therefore, we chose hDPSCs as a new source of cells for bone tissue engineering. In the current study, hDPSCs showed positive results for mesenchymal stem cell markers CD90, CD105, and CD166, and negative results for hematopoietic stem cell markers CD34 and CD45. During culture in passages 2 to 5, the pattern of positive or negative expression of specific surface proteins did not change, indicating that hDPSCs are relatively tolerant of *ex vivo* manipulation. In addition, the proliferation rate of the hDPSCs was approximately 3 times higher than that of hBMSCs^31^,^38^. Gronthos *et al.* have reported that high proliferation rate is attributed to an earlier developmental state of hDPSCs compared with hBMSCs^30^. Based on these results, hDPSCs can serve as an alternative source to supply a higher number of cells for the clinical application.

hDPSCs are multipotent stem cells that have the potential to differentiate into osteogenic cells. In the current study, we focused on the osteogenic differentiation of hDPSCs. Dexamethasone, β-glyceraldehyde-3-phosphate, l-ascorbic acid, and rhBMP-2 are the traditional efficacious osteogenic factors. Previous studies have reported the osteogenic differentiation of MSC in the presence of only osteogenic factors^39^,^40^,^41^. Our data also demonstrates that osteogenic factors (a mixture of β-glyceraldehyde-3-phosphate, l-ascorbic acid, dexamethasone, and rhBMP-2) were used successfully to induce *in vitro* osteogenic differentiation of hDPSCs, as do BMSCs^42^.

We determined that differentiated osteoblasts were characterized by ALP, ARS, and VK staining methods, which are used widely stains. The staining intensity of differentiated cells gradually increased from 1 week to 4 weeks, thereby confirming that the correct phenotype was obtained for differentiated osteoblasts. In addition, hDPSCs showed significant osteogenic differentiation ability when compared to hBMSCs in ALP assay of this study. These results have evaluated the efficacy of hDPSCs as a promising source of stem cells in bone tissue engineering.

A 3D scaffold capable of supporting bone formation is essential for neo-bone tissue regeneration^6^,^7^,^8^,^9^. Among several techniques to fabricate the scaffold, computer-designed and indirect or direct SFF fabrication technique is a recently introduced method for fabricating precisely predefined scaffold^10^,^11^,^12^. Although it can have a wide range of biomaterial selectivity, the indirect SFF fabrication technique required an additional process for printing of sacrificial 3D mold template. Thus, it needs many post-processing steps to fabricate a final scaffold^14^.

However, in this study, SFF technique yielded directly a well-defined, computer-designed, three-dimensional scaffold that was not only highly reproducible for geometry, but also did not require organic solvents during the fabrication process. This technique may therefore be easily utilized for bone tissue engineering.

The present work selected PLGC as biomaterial for scaffold fabrication, with quick turn-around and high reproducibility using SFF. The PLGC copolymer obtained, exhibited melting temperatures of 100–120 °C and 40–65 °C attributable to PLLA and PCL, respectively. Thus, the PLGC copolymer easily melted in the heating jacket, at 130 °C during the SFF method in this study. The melted PLGC copolymer exhibited easy mobility from the micronozzle and solidification at ambient temperature; therefore, it was easily plotted layer by layer.

The optimal pore size for bone tissue engineering is still controversial as there have been conflicting reports^43^. In several works, the pore sizes of tissue engineered scaffolds for osteoblast activity have been examined as about 20 to 1500 μm. It has been reported that the effective pore size for bone tissue engineered scaffolds is 200–400 μm^44^. Moreover, our unpublished data demonstrated that cells can attach and proliferate on the scaffold even with 100-μm pore size. The pore sizes of SFF fabricated scaffolds can be controlled to induce better bone regeneration under the physiological and anatomical environment. Thus, the PLGC scaffold in this study was fixed at 200-μm pore size.

In addition, the compressive modulus of the PLGC scaffold (49.3 ± 9.1 MPa) were very close to that of human’s cancellous bone (Young’s modulus of 50 MPa)^45^. We thus achieved the goal of a computer-designed, solvent-free, PLGC scaffold fabrication with quick turn-around and high reproducibility using SFF.

In this work we used a surgical bone-defect model to assess the potential of the PLGC scaffold/hDPSCs/OF in the comparison with PLGC scaffold alone, because several studies have already reported that scaffold in the absence of OF did not induce a sufficiently osteogenic differentiation for MSCs *in vivo*^46^,^47^. Moreover, our unpublished data also demonstrated that scaffold with only MSCs in the absence of OF regenerated low quantity of new bone.

The eventual goal addressed by this work was *in vivo* bone regeneration using the computer-designed, solvent-free PLGC scaffold with hDPSCs/OF. Cranial bone defect was determined as 6 mm diameter in order to safely create circular skull defect without touching the coronal or sagittal suture, although the critical size is 8 mm in rats. To confirm that it worked, we evaluated the 6 mm defect by micro-CT and histology after 4, 8, and 12 weeks. Even after 12 weeks, micro-CT showed little evidence of new bone formation with PLGC scaffold alone. In contrast, defective bone treated with PLGC scaffold with hDPSCs/OF showed clear evidence of significant neo-bone generation. The intensity from micro-CT showed a steady increase in neo-bone formation over time, reaching 51% for neo-bone at 12 weeks.

Histological images of removed bone sections were reconstructed to evaluate the degree of a neo-bone formation between transverse sections of MTS staining. We saw no definitive evidence of neo-bone formation on or extending through the PLGC scaffold alone, from either proximal or distal bone fragments.

Collagen matrix deposition was localized on the PLGC scaffold with hDPSCs/OF, indicative of neo-bone formation. In histologic images, PLGC scaffold with hDPSCs/OF showed obvious thicker bone formation at centers than in the margins of the defect. Particularly, PLGC scaffold with hDPSCs/OF showed greater quantity of the newly formed bone than that observed in the PLGC alone.

Neo-bone formation of PLGC scaffold with hDPSCs/OF occurred mostly at the lamina interna in the bottom of the implanted scaffold and upper side of the dura mater, as observed by others^48^,^49^,^50^. This was probably attributed to the better nutrition and blood supply from the dura mater side compared to the scalp side. In addition, the bottom regions of the implanted scaffold had more pronounced new bone formation than did the inner regions of the scaffold, which was likely due to limited diffusion of nutrition and blood supply toward the inside of the scaffold, as observed by others^51^. The neo-bone formation in semi-quantitative measurements reached ~54% at 12 weeks. The results for neo-bone formation were consistent in both, micro-CT and histological evaluations.

Furthermore, we investigated if *in vivo* neo-bone formation occurs in response to scaffold degradation. In conventional methods, scaffold degradation would be determined from sacrificed animals at each time point. In this work, we captured images using fluorescent-labeled PLGC scaffold as well as GPC and NMR measurement of PLGC scaffold obtained from sacrificed animals for comparison.

We confirmed that SFF fabricated PLGC scaffold degraded gradually over the appropriate experimental time frame and the degradation value showed a good relationship between fluorescent intensity and molecular weight changes in the scaffold. In addition, we observed that the ingrowth of bone tissue gained from the results of the micro-CT and histology increased gradually and correlated with the scaffold degradation.

## Conclusion

This study was performed to investigate potential neo-bone formation using a computer-designed and solvent-free PLGC scaffold with hDPSCs. hDPSCs served as a promising source of stem cells for bone tissue engineering and differentiated into osteoblasts in the presence of osteogenic factors. The PLGC scaffold with hDPSCs showed evidence of neo-bone formation at the defect site. The gradual ingrowth of bone tissue observed by micro-CT and histology showed a correlation with scaffold degradation. In conclusion, our results show that a computer-designed and biodegradable PLGC scaffold with hDPSCs effectively supports neo-bone formation.

## Methods

### Culture and characterization of hDPSCs

Fresh hDPSCs were isolated from third molar teeth that had been extracted from healthy, nonsmoking adults (aged 22 years) at Inha Hospital, Korea. The protocols of this study were approved by Internal Review Board for Human Subjects Research and Ethics Committee of Inha Hospital (Approval No. IUH IRB 12–150), and informed consent was obtained from the subjects before enrollment in this study.

The protocols of this study were carried out in accordance with the approved guidelines. hDPSCs were cultured in a 75-cm^2^ tissue culture flask (BD Falcon; CA, USA) in α-minimal essential medium (α-MEM; Gibco; NY, USA) containing 15% fetal bovine serum (FBS; Gibco; NY, USA), 2 mmol/L l-glutamine (Gibco; NY, USA), 100 μmol/L ascorbic-acid-2-phosphate (Sigma; MO, USA), and 1% penicillin-streptomycin (PS; Gibco; NY, USA) in 5% CO_2_ at 37 °C. Fifth-passage hDPSCs were used for the bone tissue engineering experiments. For identification of hDPSCs, the phenotype of hDPSCs was determined by flow cytometry using a FACScan cytometer (BD Bioscience; CA, USA). CD90, CD105, and CD166 cell-surface antigens were considered as positive markers, while CD34 and CD45 were considered as negative markers. The hDPSCs expressed mesenchymal stem cell markers, CD90 (>99.3%), CD105 (>95.6%), and CD166 (99.4%), while they showed no expression of the hematopoietic stem cell markers CD34 (<0.01%) and CD45 (<0.01%). To measure the proliferation of hDPSCs as a potential source of cells for bone tissue engineering, the number of hDPSCs and human bone marrow stem cells (hBMSCs, Pharmicell, Seoul, Korea) for comparison was counted after cultivation for 6 days at passages 2 through 6.

### *In vitro* osteogenic differentiation of hDPSCs

The hDPSCs (1 × 10^4^) were seeded into 6-well plates (Nunc; Roskilde, Denmark) and incubated for 24 h. The seeded hDPSCs were treated with α-MEM containing 10% FBS, 10 mM β-glyceraldehyde-3-phosphate (Sigma; MO, USA), 60 μg/mL l-ascorbic acid (Sigma; MO, USA), 10 nM dexamethasone (Sigma; MO, USA), and 40 ng rhBMP-2 (Cellumed; Seoul, Korea) as osteogenic factors (OF) for *in vitro* osteogenic differentiation and α-MEM containing 10% FBS as a negative control. To confirm osteogenic induction using hDPSCs, alkaline phosphatase (ALP), Alizarin red S (ARS), and Von Kossa (VK) staining was carried out after culture for 1, 2, 3, and 4 weeks.

For ALP staining, the hDPSCs were washed twice with phosphate-buffered saline (PBS) and fixed with 4% paraformaldehyde (Biosesang; Gyenggi, Korea). The staining procedure was performed using a Leukocyte Alkaline Phosphatase Kit (Sigma; MO, USA) with naphthol AS-BI, following the manufacturer’s protocol.

For ARS staining, the cells were washed twice with PBS and fixed in 4% paraformaldehyde. The fixed cells were washed thrice with deionized water and incubated with 0.2% ARS solution for 15 min. Then, the cells were washed and observed with an optical microscope (Carl Zeiss Microimaging GmbH; Göttingen, Germany).

For VK staining, the fixed cells were washed thrice with deionized water and treated with 5 wt% silver nitrate (Sigma; MO, USA) for 1 h. The color of the stained cells was developed with a sodium carbonate/formalin solution for 1 min. Counter stains of VK and Nuclear Fast Red (Sigma; MO, USA) were used for the nucleus.

### Determination of *in vitro* ALP contents of differentiated hDPSCs and hBMSCs

To compare differentiation ability between hDPSCs and hBMSCs, cells were seeded in 24-well tissue culture plates with density of 2 × 10^4^ cells/well simultaneously and incubated for 24 h in 37 ^o^C, 5% CO_2_ condition. After 24 h, hDPSCs and hBMSCs were cultured with OF for osteogenic differentiation. After 1 and 7 days, ALP contents were measured using SensoLyte^®^ pNPP Alkaline Phosphatase ELISA Assays Kit (ANASPEC; CA, USA) for end-point reading following manufacturer’s protocol. The cells were trypsinized and treated with 1 × assay buffer containing 0.2% Triton X-100 for 10 min in 4 ^o^C under shaking. The samples were centrifuged at 2500 g for 10 min at 4 ^o^C and the supernatant was used for detection of ALP.

### Characterization

^1^H nuclear magnetic resonance (NMR) spectra of the PLGC copolymer were measured using a Varian Mercury Plus 400 system with an internal standard of CDCl_3_ in the presence of tetramethylsilane (TMS). Molecular weight distributions of the PLGC copolymer were measured using a YL-Clarity GPC system (YL 9170 RI detector) with three columns (Shodex K-802, K-803, and K-804 polystyrene gel columns) at 40 °C. For this measurement, polystyrene calibration was performed and CHCl_3_ was used as an eluent at a flow rate of 1.0 mL/min. The melting temperature (*T*_m_) of the PLGC copolymers was determined by differential scanning calorimetry (DSC; Q 1000, TA Instruments; Alzenau, Germany) from −80 to 200 °C at a heating rate of 5 °C/min under an atmosphere of nitrogen.

### Synthesis of PLGC copolymers

MPEG, with a number-average molecular weight (*M*_n_) of 750 g/mol (Aldrich; MO, USA) and stannous octate (Aldrich; MO, USA) were used as received. ε-Caprolactone (CL) was distilled over CaH_2_ under reduced pressure. l-lactide (LA) and glycolide (GA) (Boehringer Ingelheim; Ingelheim, Germany) was recrystallized twice in ethyl acetate. All glasses were dried by heating in vacuum and were handled under a dry nitrogen stream. MPEG (0.012 g, 0.016 mmol) and toluene (60 mL) were introduced into a flask. Water was removed from the MPEG solution by azeotropic distillation before toluene was distilled off to give a final volume of 10 mL. LA (5.2279 g, 36.0 mmol), GA (0.7021 g, 6.0 mmol), and CL (2.07 g, 18.1 mmol) were added to the MPEG solution at room temperature under an atmosphere of nitrogen, and then 0.2 mL stannous octate (0.1 M solution in dried toluene) was added to the solution. After stirring at 160 °C for 24 h, the reaction mixture was poured into a mixture of *n*-hexane and ethyl ether (*v/v *= 4/1) to precipitate the polymer, which was separated from the supernatant by decantation, redissolved in CH_2_Cl_2_, and then filtered. The resulting solution was concentrated by rotary evaporation and dried in a vacuum to yield a colorless polymer. The molecular weights of the PLLA, PGA, and PCL segments in the block copolymers were determined by ^1^H NMR spectrometry by comparing the intensity of the methylene proton signals of MPEG (standard, 750 g/mol), PLLA, PGA, and PCL at δ of 5.2, 4.7, 4.1, and 2.4, respectively.

### Synthesis of PLGC copolymers with fluorescein isothiocyanate (FITC) (PLGC -FITC)

PLGC (4 g, 0.008 mmol) and toluene (80 mL) were introduced into a flask. Water in the PLGC solution was removed by azeotropic distillation. Toluene was distilled off to give a final volume of 30 mL. FITC (3.7 mg, 0.01 mmol) was added to the PLGC solution at room temperature under a nitrogen atmosphere. Subsequently, 0.1 mL stannous octate (0.1 M solution in dried toluene) was added. After the reaction mixture was stirred at 130 °C for 24 h, it was poured into a mixture of *n*-hexane, ethyl ether, and methanol (*v/v/v *= 2/1/1) to precipitate a polymer, which was separated from the supernatant by decantation, redissolved in CH_2_Cl_2_, and then filtered. The resulting solution was concentrated by rotary evaporation and then dried in vacuum to give an 87% yield of PLGC-FITC.

### Fabrication of the circular PLGC scaffold

The SFF 3D plotter (ProtekKorea; Daejeon, Korea) consisted of a plotting system with a heating jacket and a stainless steel cylinder with a micronozzle (internal diameter, 300 μm) moved by an air dispenser in the direction of the *x*–*y*–*z* stage. The plotting system was controlled by Scaffold Path Generation SW computer software (Korea Institute of Machinery and Materials; Daejeon, Korea). PLGC was added and melted at 130 °C in the stainless steel barrel of the heating jacket. The melted PLGC was extruded from the micronozzle with an air pressure of 100 kPa. The computer-designed circular PLGC scaffold (5 mm diameter × 1 mm length) was fabricated using layer-to-layer orthogonal orientation, with 200-μm pores in the vertical cross-section. PLGC was deposited into a series of parallel lines along the *y* direction in the first layer parallel to the *x* direction in the second layer; the same deposition procedure was applied to the third and fourth layers. The fabrication process produced a solidified PLGC scaffold under normal ambient temperature. The compressive modulus of PLGC scaffold was tested using an Instron mechanical tester (H5K-T UTM, Tinius Olsen, PA, USA). The load was applied parallel to the perpendicular axis of the PLGC scaffold. The value of the compressive modulus was determined with a 50% compression of the PLGC scaffold. The fabricated PLGC scaffold was also examined by scanning electron microscopy (SEM) using a JSM-6380 SEM (JEOL, Tokyo, Japan).

### Cell attachment and proliferation assays on fabricated PLGC scaffold

For cell culture experiments, the fabricated PLGC scaffold were placed individually into the wells of a 24-well tissue culture plate (Falcon, USA) and then incubated for 1 h under culture media. After the suction of media, the hDPSCs (1 × 10^5^) were transferred to each PLGC scaffold. The culture media (described in previous section) was changed every 3 days throughout the studies. At each time point, the PLGC scaffolds were washed three times with PBS and fixed with 4% formaldehyde for 24 h. Then the scaffolds were washed 2 times with PBS and 0.1% triton X-100 in PBS was added to each well for 5 minutes. After washing with PBS, 1% bovine serum albumin was added for 30 minutes and 50 μL staining solution (Alexa Fluor® 594 Phalloidin; Molecular probe, Eugene, OR, USA) diluted in 1 mL PBS was added for 30 minutes. Then the PLGC scaffolds were washed 2 times with PBS and counterstained with 4’,6’-diamidino-2-phenylindole hydrochloride (DAPI). Finally the scaffolds were washed with DW and dried at room temperature.

Fluorescence images were visualized under an LSM 710 (Carl Zeiss Microimaging GmbH, Göttingen, Germany) and analyzed with ZEN 2009 software (Carl Zeiss Microimaging GmbH, Göttingen, Germany). SEM was used to examine hDPSCs on fabricated PLGC scaffold. The PLGC scaffold after culturing for 1, 4 and 7 days and fixed with 2.5% glutaraldehyde for 24 h, followed by ethanol dehydration. The fixed PLGC scaffold was coated with a conductive layer of gold using a plasma-sputtering apparatus (Emitech, K575, Kent, UK), and scanning electron microscopy (Stereoscan 440, Leica Cambridge, Germany) images were obtained.

### Animal implantation surgery

The protocols of this study were approved by the Institutional Animal Experiment Committee (Approval No. 2012-0004) at Ajou University School of Medicine. Bone tissue engineering experiments using Sprague–Dawley rats (weight, 320–350 g; age, 8 weeks) were carried out in accordance with the approved guidelines. Each rat was anesthetized using zoletil and rompun (1:1 ratio, 1.5 mL/kg).

A Total of 30 SD rats were randomly assigned into the two experimental groups: PLGC scaffold alone (*n *= 15) and PLGC scaffold with hDPSCs/OF (*n *= 15). A full-thickness circular hole, 6 mm in diameter, was created in the cranial bone of each rat and the circular PLGC scaffold alone was inserted into the hole. The detect sizes was determined as 6 mm in order to safely create circular skull defect without touching the coronal or sagittal suture. For implantation of PLGC scaffold with hDPSCs/OF, PLGC scaffold were placed individually into the wells of a 24-well tissue culture plate (Nunc; Roskilde, Denmark) and then incubated for 1 h under culture media. After the suction of media, hDPSCs (1 × 10^6^) were transferred on PLGC scaffold and incubated for 4 h in 5% CO_2_ at 37 °C. Then, the hDPSCs-loaded PLGC scaffold was implanted at the cranial defect. After that, 20 μL of OF consisting of 10 μL α-MEM containing 10% FBS, 300 mM β-glyceraldehyde-3-phosphate, 1.8 mg/mL L-ascorbic acid, 300 nM dexamethasone, and 4 μg of rhBMP-2 (Cellumed; Seoul, Korea) added inside the implanted hDPSCs-loaded PLGC scaffold.

The PLGC scaffold alone and PLGC scaffold/hDPSCs/OF was allowed to develop and then biopsied at different time points over 4, 8, and 12 weeks. At each of the post-implantation time points (4, 8, and 12 weeks), the rats (*n *= 5 for each group) were sacrificed and the PLGC implant sites were removed individually.

For the *in vivo* biodegradation experiment, the removed PLGC scaffold alone and PLGC scaffold/hDPSCs/OF was placed in a test tube, to which CH_2_Cl_2_ (1 mL) was added to dissolve the PLGC portion of the implant and 1 mL distilled water was added to solubilize the tissue. The resulting mixture was sonicated for 90 min at 25 °C and then centrifuged at 10,000 rpm for 5 min. The CH_2_Cl_2_ solution was collected, CH_2_Cl_2_ was removed, and the remaining PLGC-containing degraded compounds were freeze-dried until the residue reached a constant weight. The characteristic peaks of degraded compounds were measured by NMR. The molecular weights of the *in vivo*-degraded PLGC scaffold without or with hDPSCs/OF were determined at the maximum GPC peak and by NMR. The mass changes of *in vivo*-degraded PLGC scaffold was calculated by the relative ratio of molecular weights of degraded polymer (6 or 8) and parent MPEG (1) (in Fig. 4d) determined during the experimental 12 weeks and on the initial day.

### *In vivo* fluorescence imaging

At 4, 8, and 12 weeks, the PLGC-FITC scaffold implanted in the rat cranial bone was removed and its image was captured at a 515 nm wavelength (excitation wavelength, 470 nm) using a fluorescence imaging system (FO ILLUM PL-800; Edmund Optics; NJ, USA; 150W EKE Quartz Halogen light; glass reference number OG515 filter). After digitization using a charge-coupled device, fluorescence images were visualized with the Axiovision Rel. 4.8 software. The intensity of fluorescence images was determined using ImageJ software, version 1.44 (National Institutes of Health; Bethesda, MD, USA).

### Microcomputed tomography (micro-CT)

The neo-bone formation was analyzed by micro-CT, carried out with a Skyscan 1076 (SKYSCAN; Konitch, Belgium) device, at 36.44 pixel resolution and 80 ms exposure time with an energy source of 40 kV and a 250 mA current. Approximately 180 projections were acquired over a rotation range of 180°, with a 4° rotation step. The full length of each bone was scanned, and, on average, consisted of 850 slices. Three-dimensional virtual models of representative regions in the scaffolds were created and visualized using MIMICS 16.0 software (Materialise’s interactive medical image control system; Leuven, Belgium). The visualized 3D images were shown in the gross profiles including trabecular and cortical bone ranges. The density of newly formed bone was determined by assigning a threshold for total bone mineral content within the initial defect and subtracting any contribution from the scaffold. The extent of the neo-bone formation in micro-CT was calculated by the relative ratio of newly formed bone mineral density determined during the experimental 12 weeks and on the initial day.

### Histological analysis

On weeks 4, 8, and 12 after implantation, the rats were sacrificed and the implants were individually dissected and removed from cranial bone. The implants were immediately fixed with 10% formalin and decalcified using decalcifying solution-lite (Sigma; MO, USA) for 8 h. The fixed tissues were dehydrated and embedded in paraffin. The embedded specimens were sectioned (4 μm) and the sections were stained with hematoxylin and eosin (H&E), VK, and Masson’s trichrome stains.

For H&E staining, the paraffinized slides were depaffinized with xylene 2 times and hydrated using 100, 95, 80, 70, and 60% of ethyl alcohol in regular sequence. Then, samples were washed in running tap with water and stained with hematoxylin and eosin for 3 min respectively. Thereafter, stained slides were fixed and mounted with mounting medium (Muto Pure Chemicals; Tokyo, Japan).

For VK staining, the slides were washed thrice with deionized water (DW) and treated with 5 wt% silver nitrate (Sigma; MO, USA) solution for 1 h, after which the slides were washed with DW and the color of the stained tissues was developed with a sodium carbonate/formalin solution for 1 min. The nucleus of the tissues was stained with Nuclear Fast Red (Sigma; MO, USA).

For Masson’s trichrome staining, the slides were incubated for 15 min at 56 °C in Bouin’s solution (Sigma; MO, USA) and then washed under a running tap to remove excess stain. The nucleus of the specimens was stained with Weigert’s iron hematoxylin (Sigma; MO, USA) for 5 min. The slides were washed in running tap water for 5 min and rinsed in DW. The slides were placed in a phosphotungstic/phosphomolybdic acid solution for 5 min and then stained with Aniline Blue solution for 5 min. The slides were treated with 1% acetic acid for 2 min and then fixed with mounting medium (Muto Pure Chemicals; Tokyo, Japan). The traverse sections of MTS staining was visualized and semi-quantitatively measured by using Image J 1.44 (National institute of Health, MD, USA) at each experimental period. The extent of the neo-bone formation was calculated by the comparing the ratio of positive MTS area and defect area.

The areas of H&E, VK and MTS histology imaging assignable to PLGC scaffold were calculated to determine the volume changes of *in vivo*-degraded PLGC scaffold. The extent of the degraded PLGC scaffold was calculated by the comparing the ratio of the area assignable to non-degraded PLGC scaffold and the defect area during the experimental period and on the initial day.

### Statistical analysis

Cell numbers of hDPSCs and hBMSCs were counted three times (*n *= 3) at each time and passage. ALP contents and cytotoxicity of hDPSCs were conducted using 3 samples for each group. Fluorescence intensity of PLGC-FITC scaffold, MTS staining positive area and the data obtained in CT images were calculated 3 times using Image J program. All data are presented as the mean and standard deviation (SD). The results were analyzed with one-way analysis of variance (ANOVA) with Bonfferoni correction using SPSS 12.0 software (SPSS Inc.; IL, USA).

## Additional Information

**How to cite this article**: Yeon Kwon, D. *et al.* A computer-designed scaffold for bone regeneration within cranial defect using human dental pulp stem cells. *Sci. Rep.* **5**, 12721; doi: 10.1038/srep12721 (2015).

## Supplementary Material

Supplementary Information

## Figures and Tables

**Figure 1:**
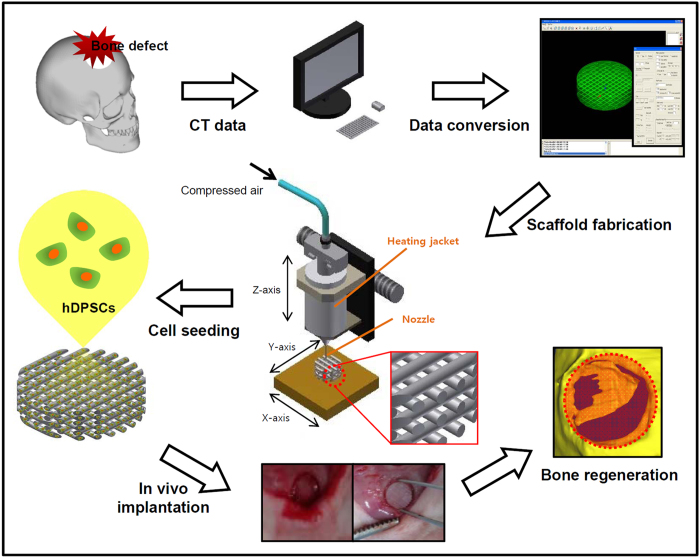
Schematic showing the experimental procedures using an SFF system for neo-bone formation. (A skull image was drawn by D.Y.K. and S.H.J. using software of Autodesk Inventor 2012 (Autodesk, NC, USA)).
