# Supplementary material for: A computer-designed scaffold for bone regeneration within cranial defect using human dental pulp stem cells
Source: Sci Rep. 2015 Aug 3;5:12721. doi: 10.1038/srep12721 (PMC4522608; doi:10.1038/srep12721)
Supplement: Supplementary Information [file srep12721-s1.doc]

***Supplementary information***

**A computer-designed scaffold for bone regeneration within cranial defect using human dental pulp stem cells**

Doo Yeon Kwon, Jin Seon Kwon, Seung Hun Park, Ji Hun Park, So Hee Jang, Xiang Yun Yin, Jeong-Ho Yun, Jae Ho Kim, Byoung Hyun Min, Jun Hee Lee, Wan-Doo Kim, Moon Suk Kim*


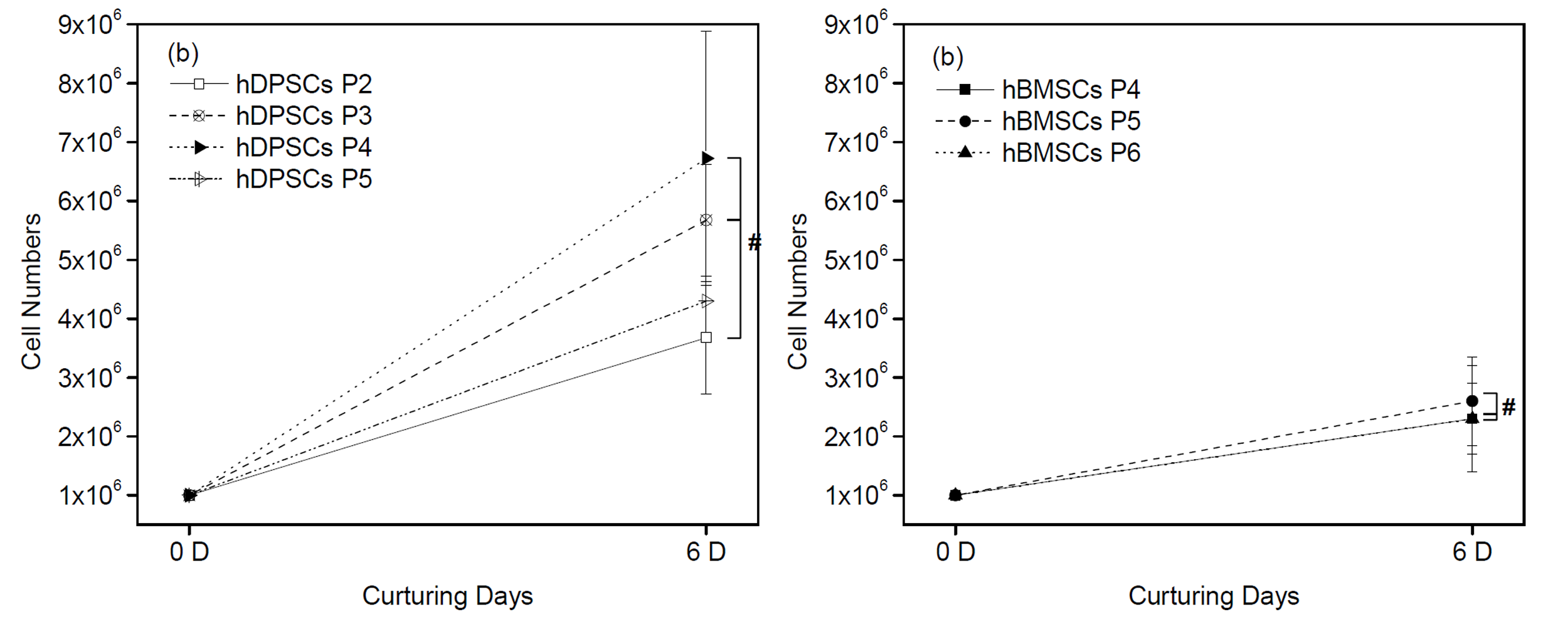


**Figure S1**. Cell numbers of (a) hDPSCs and (b) hBMSCs for 6 days in each passage. (^#^*p* > 0.5)


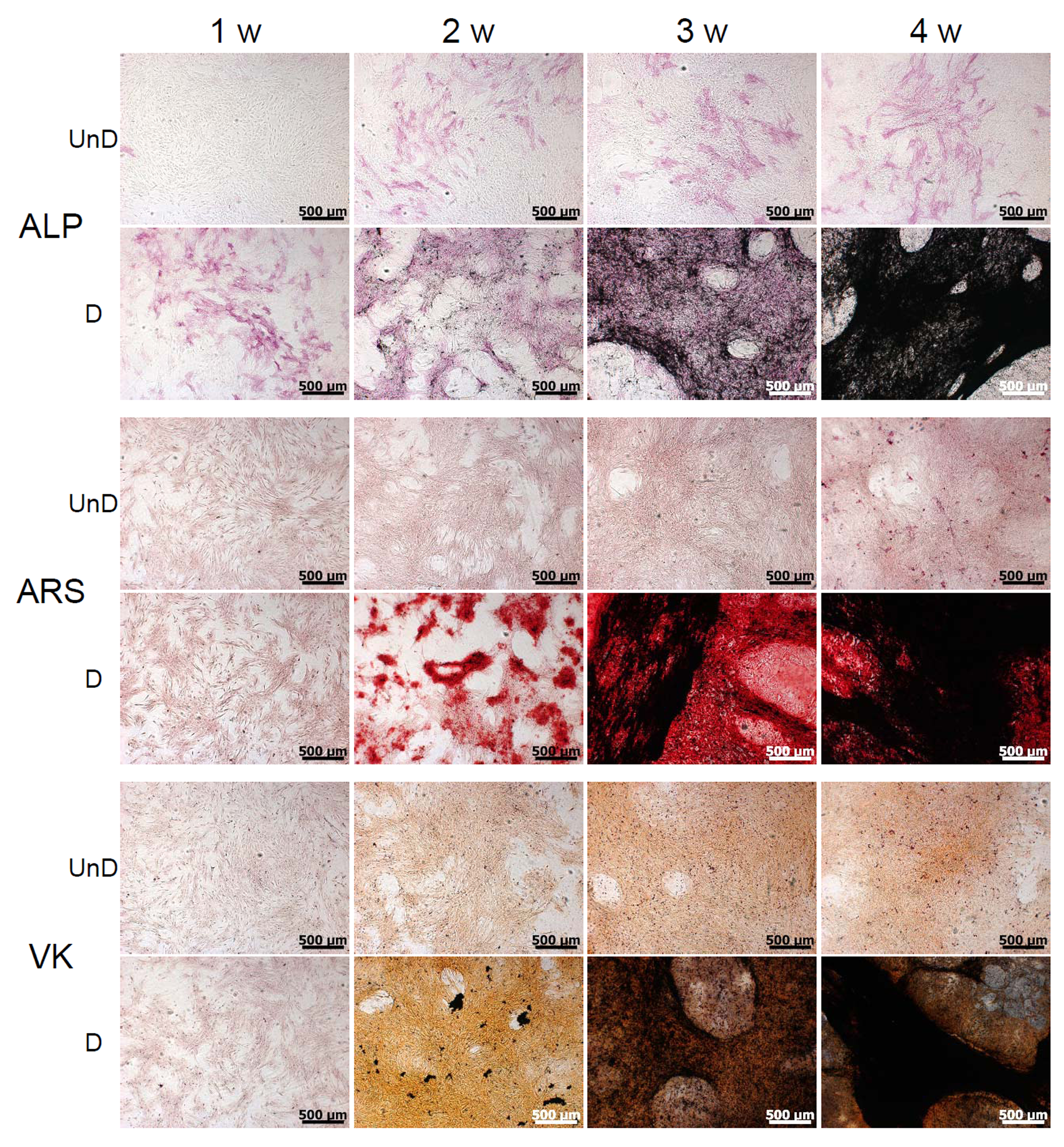


**Figure S2**. Images of undifferentiated hDPSCs in non-osteogenic medium (upper) or differentiated hDPSCs in osteogenic medium (lower) stained with alkaline phosphatase (ALP), Alizarin Red (ARS), and Von Kossa (VK) (magnification 50×, scale bars represent 500 μm). (Abbreviations: UnD, Undifferentiated hDPSCs; D, Differentiated hDPSCs)


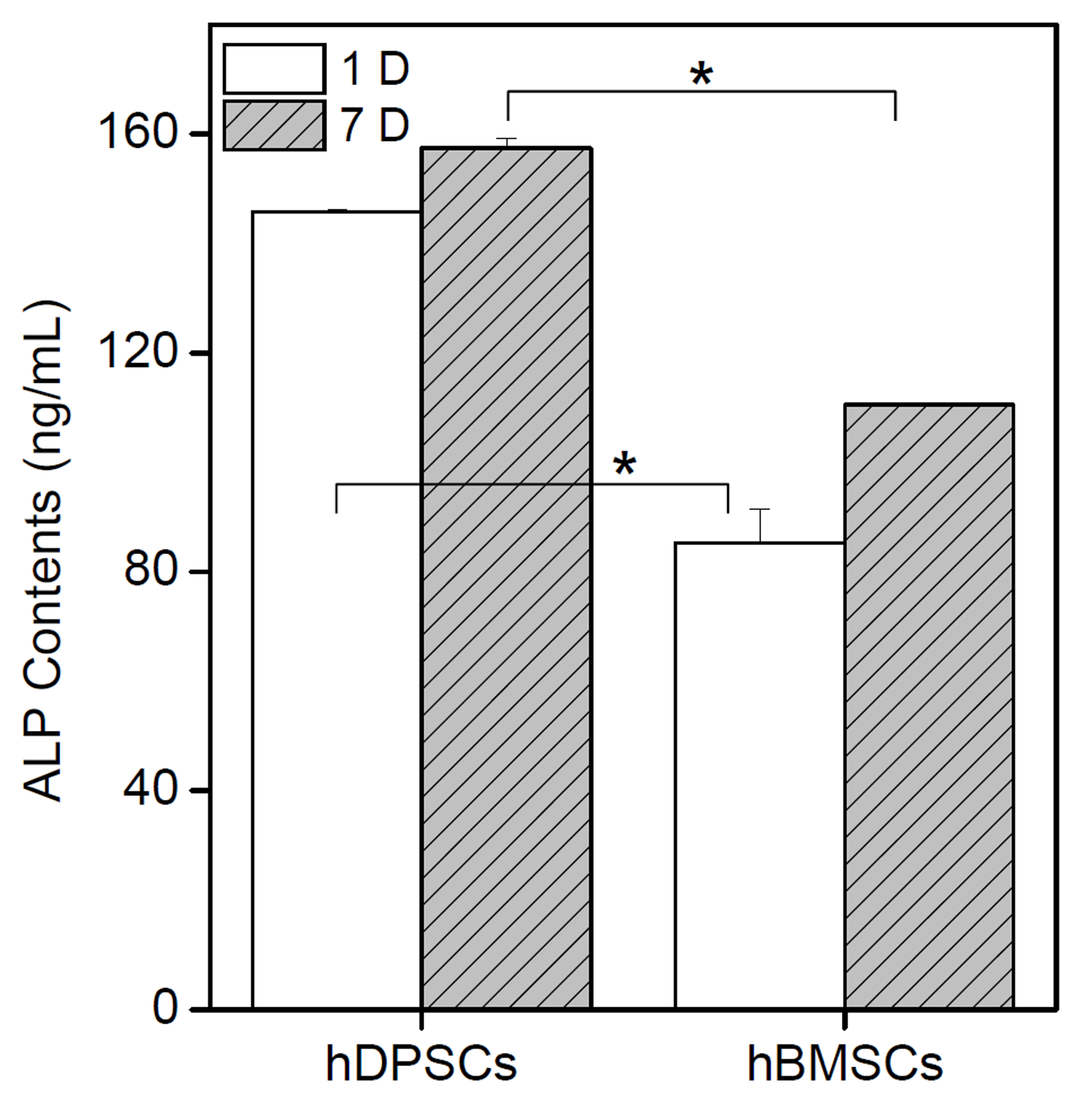


**Figure S3**. *In vitro* ALP contents of differentiated hBMSCs and hDPSCs at 1 and 7 days. (**p* < 0.001).


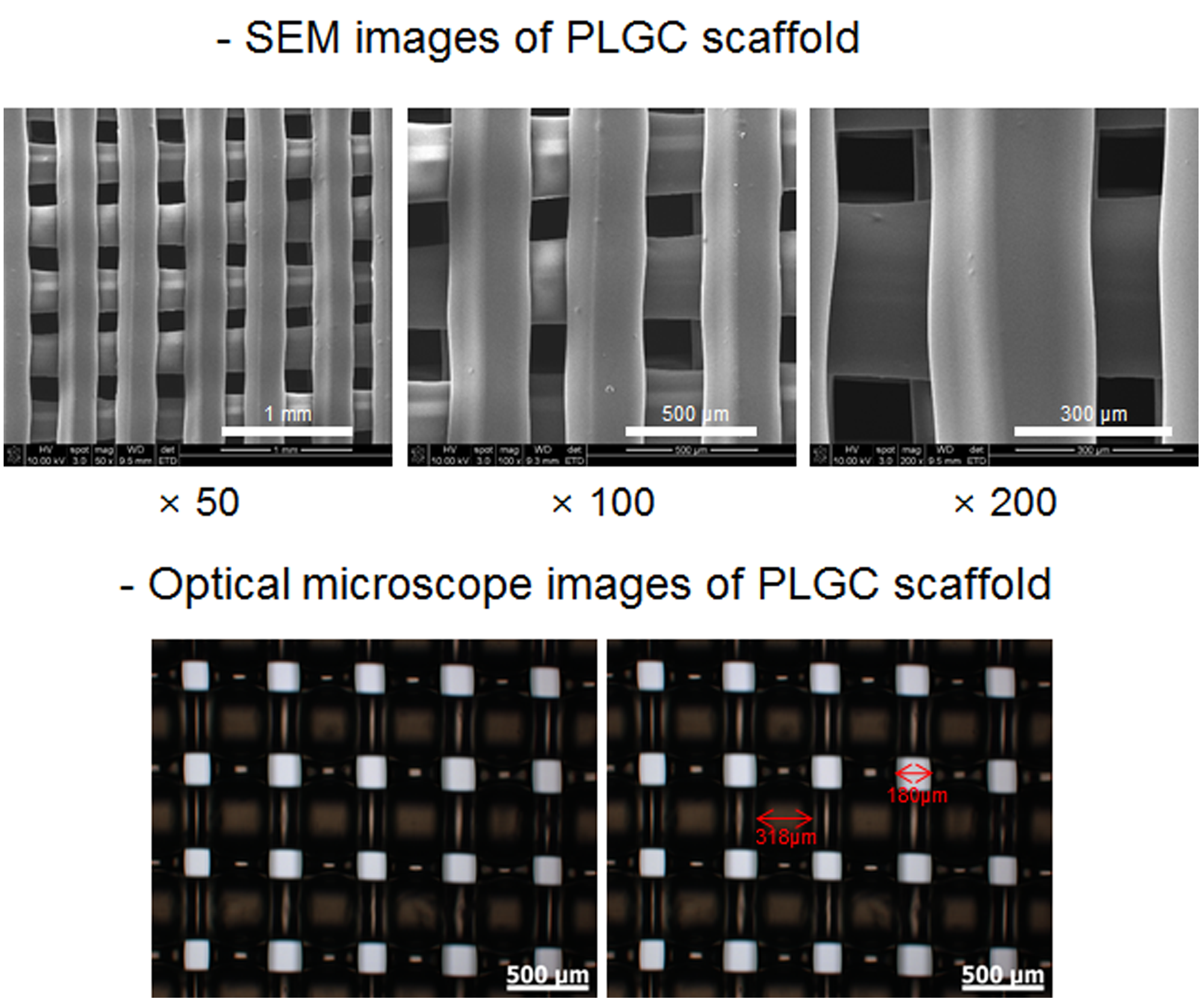


**Figure S4**. SEM and optical images of PLGC scaffold.


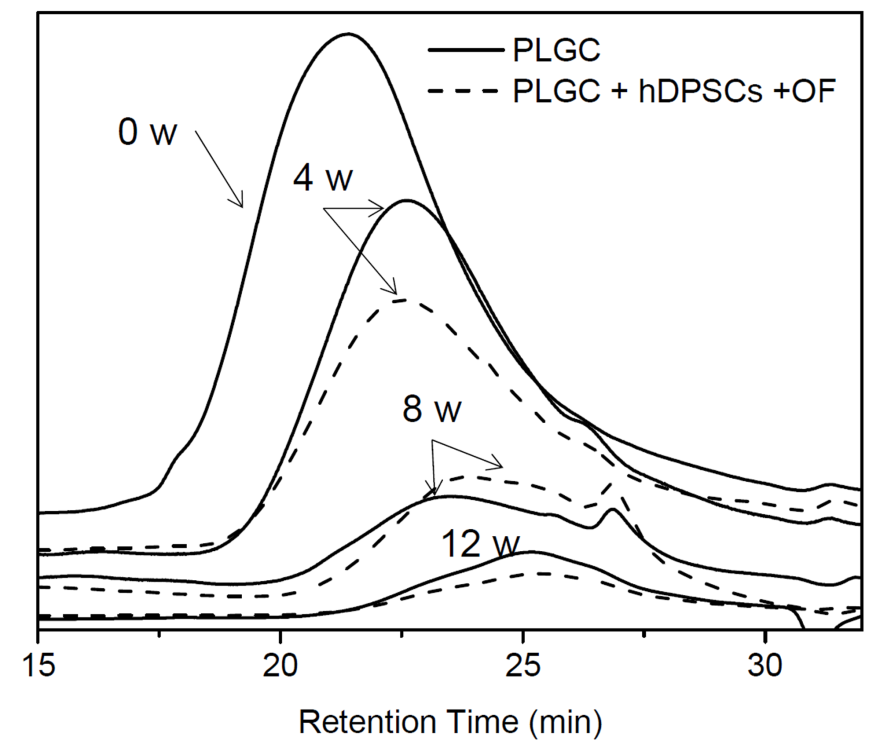


**Figure S5**. GPC peaks of *in vivo* degraded PLGC scaffold without or with hDPSCs/OF for 12 weeks.


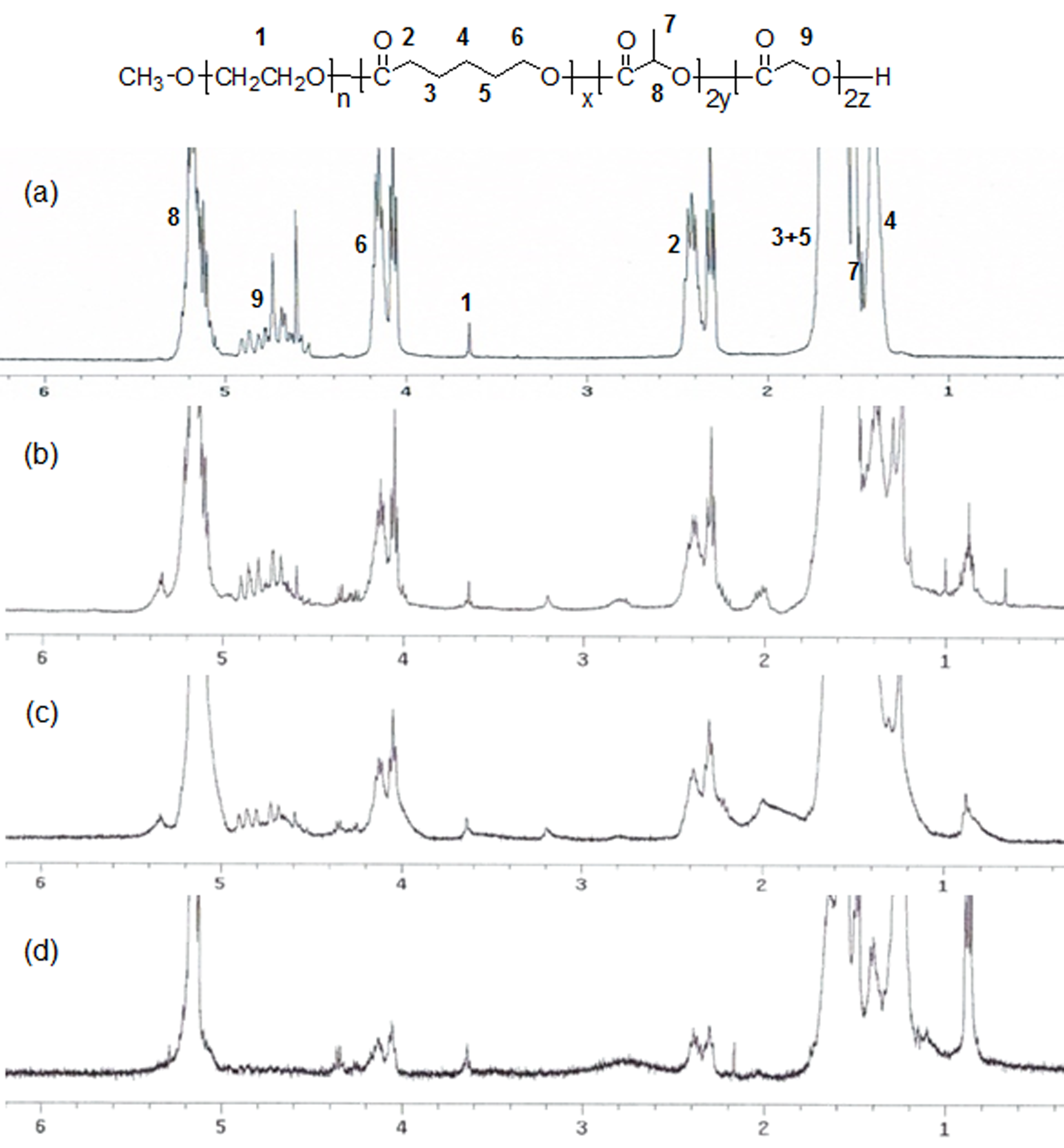


**Figure S6**. ^1^H NMR spectra (a) before degradation, and of *n*-hexane and ethyl ether insoluble portions of the *in vivo* degraded PLGC scaffold after (b) 4, (c) 8, and (d) 12 weeks.

**Figure S7**. Bone regeneration determined from micro-CT of rats receiving the PLGC scaffold without or with hDPSCs/OF at 0–12 weeks after implantation.


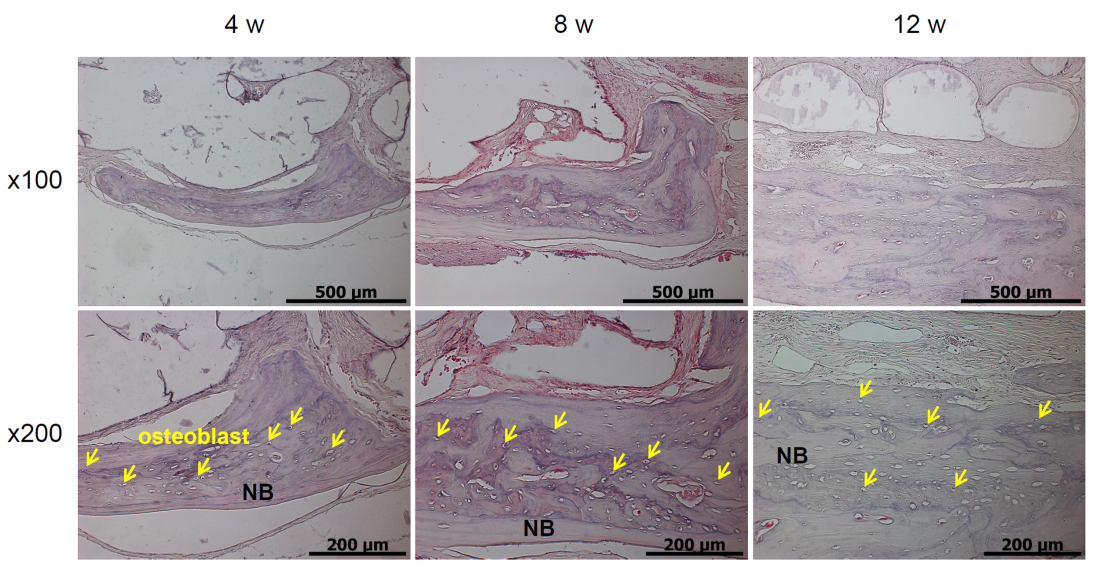


Figure S8. Enlarged H&E staining of cranial bone for the PLGC scaffold with hDPSCs/OF at 4, 8 and 12 weeks after implantation. (NB indicate neo-bone)


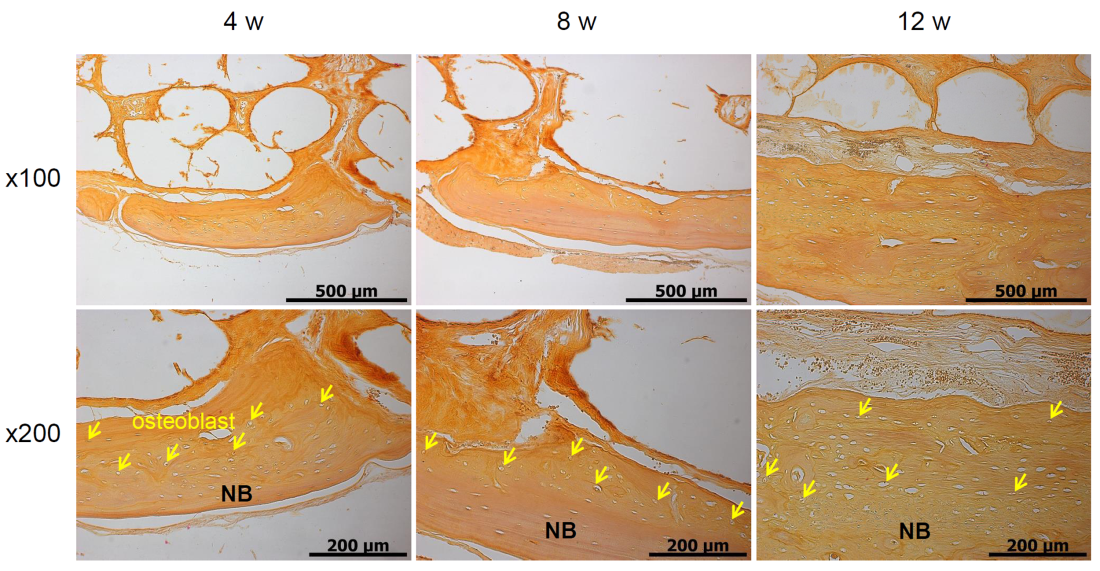


Figure S9. Enlarged von-Kossa staining of cranial bone for the PLGC scaffold with hDPSCs/OF at 4, 8 and 12 weeks after implantation. (NB indicate neo-bone)


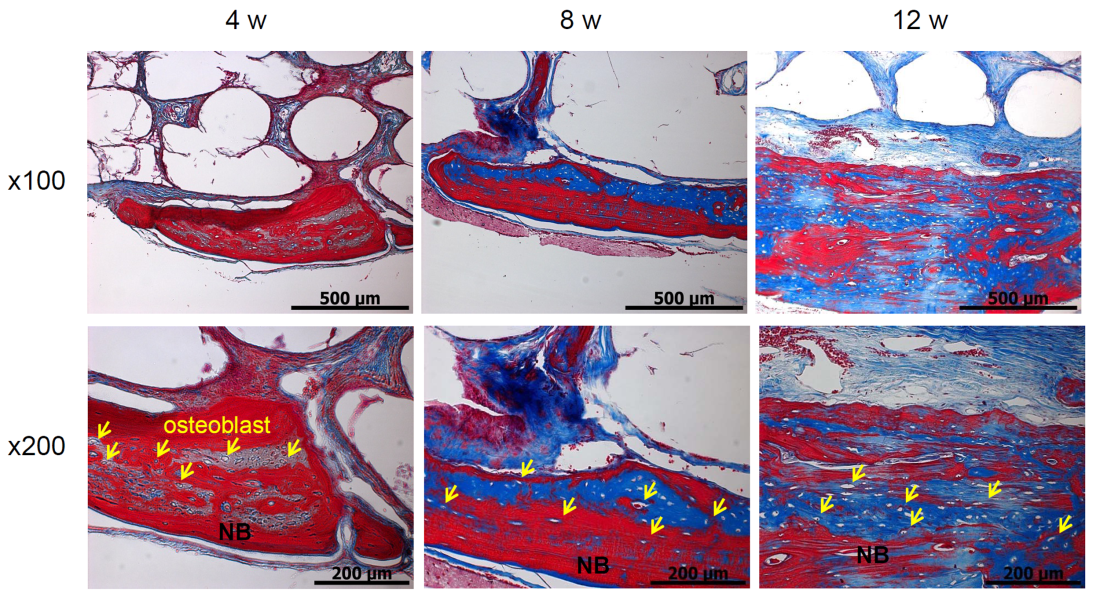


Figure S10. Enlarged Masson’s trichrome staining of cranial bone for the PLGC scaffold with hDPSCs/OF at 4, 8 and 12 weeks after implantation. (NB indicate neo-bone)

**
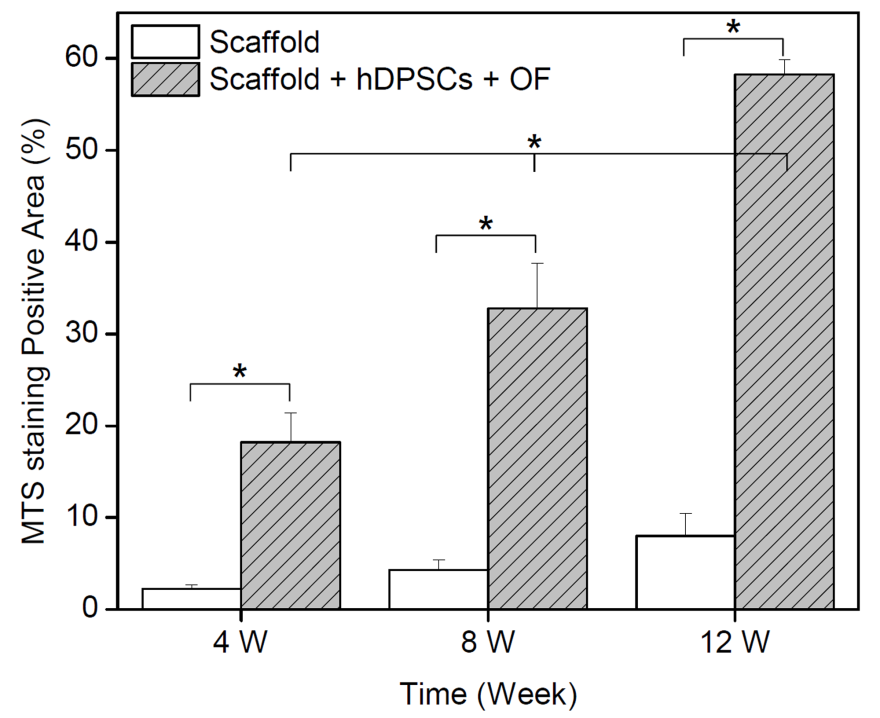
**

**Figure S11**. Masson’s trichrome-positive cells for the PLGC scaffold without or with hDPSCs/OF at 4, 8 and 12 weeks after implantation were counted on 3 images from each slide, and counts were averaged within each group. (**p* < 0.001)

**Figure S12**. The extent of remained scaffold areas was determined by histology images of H&E, VK and MTS staining and ^1^H NMR at 0–12 weeks after implantation.
